# The State of the Art in mobile Augmentative and Alternative Communication Devised for People with Dementia: Evaluation of Features and Functions

**DOI:** 10.1002/alz.094244

**Published:** 2025-01-09

**Authors:** Mariateresa (Teri) H Muñoz, Joana Okine, Ellen L Brown, Nicole Ruggiano

**Affiliations:** ^1^ Florida International University, Miami, FL USA; ^2^ University of Alabama, Tuscaloosa, AL USA; ^3^ UAB Alzheimer’s Disease Center, Birmingham, AL USA

## Abstract

Patients with dementia (PWD) often face challenges with daily activities due to communication challenges, which may lead to negative outcomes for those with dementia and their family caregivers alike. Augmentative and alternative communication (AAC) devices have demonstrated to be feasible in supporting communication among PWD (May et al., 2019) through text, graphics, and/or sound. Traditional AAC devices have been non‐electronic memory and communication aids (May et al., 2019). However, in recent years many primary caregivers of PWD are seeking out mobile applications to address this challenge using their smartphone or tablet. However, mobile applications addressing communication skills specifically for dementia care are scarce despite their potential to improve quality of life (Ambegaonkare et al., 2021; Brown et al., 2023). To better understand the current landscape of AAC mobile applications, this systematic review identified and evaluated the features and design of commercially‐available mobile applications. A total of 27 AAC apps were identified through searches on the Apple IOS store and Google Play, after excluding duplicates and apps that targeted primarily children. Four research team members were involved in cataloging features and evaluating the apps using a modified version of the Mobile App Rating Scale (Stoyanov et al., 2015). The price of apps varied, though many listed as being free or low‐cost often required higher‐priced subscriptions or licensing fees. All apps were compatible for English speakers, though several were available in multiple languages. It was found that the apps varied in their potential for engagement, functionality, aesthetics, and information. It was also found that many apps are limited in their potential to support caregiving activities (e.g., mealtime, dressing) for those with communication barriers related to cognitive impairment. Apps that are limited in their ability to customize the interface to the user’s preferences for daily care may impede person‐centered care practices for PWD. Implications for practice, policy, and research are discussed.

